# Association Between Biofilm Phenotype and Antimicrobial Resistance in Foodborne *Listeria monocytogenes* from Northern Kazakhstan

**DOI:** 10.3390/antibiotics15090921

**Published:** 2026-09-17

**Authors:** Zulkyya Abilova, Bulat Balabayev, Pavel Shevchenko, Madina Yernazarova, Aitbay Bulashev, Raushan Rychshanova, Albina Gabitova

**Affiliations:** 1 Department of Veterinary Medicine, Faculty of Agricultural Sciences, NPLC “Akhmet Baitursynuly Kostanay Regional University”, Kostanay 110000, Kazakhstan; zulkiiaabilova@gmail.com (Z.A.); b22780550@gmail.com (B.B.); 2 Research Institute of Innovative Technologies, NPLC “Akhmet Baitursynuly Kostanay Regional University”, Kostanay 110000, Kazakhstan; pavel87011339688@gmail.com (P.S.); madinaernazarova102@gmail.com (M.Y.); 3 Department of Animal Husbandry and Biological Sciences, Institute of Animal Science and Veterinary Medicine, Seifullin Kazakh Agrotechnical Research University, Astana 010000, Kazakhstan; a.bulashev@kazatu.edu.kz

**Keywords:** *Listeria monocytogenes*, antimicrobial resistance, multidrug resistance, biofilm formation, antimicrobial resistance genes, food safety, animal-derived food products

## Abstract

**Background/Objectives:** *Listeria monocytogenes* is a major foodborne pathogen of public health concern because of the severe outcomes associated with invasive listeriosis, its ability to persist throughout the food chain, and its capacity to form biofilms. In this study, the occurrence, antimicrobial resistance, antimicrobial resistance genes, and biofilm-forming capacity of *L. monocytogenes* from food of animal origin in Northern Kazakhstan were investigated, and the association between the biofilm phenotype and antimicrobial resistance was also assessed. **Methods:** A total of 1561 samples were analyzed. *L. monocytogenes* was isolated and identified according to ISO 11290-1 and confirmed by MALDI–TOF MS. Antimicrobial susceptibility was determined by disk diffusion according to EUCAST recommendations. Resistance genes were detected by PCR, and biofilm formation was assessed using a crystal violet assay. **Results:** *L. monocytogenes* was detected in 34 (2.18%) samples. Resistance to at least one antimicrobial agent was observed in 18 (52.9%) isolates, with the highest resistance rate observed for trimethoprim–sulfamethoxazole (14/34, 41.2%). Multidrug resistance was detected in 8 (23.5%) isolates and was defined as resistance to at least three antimicrobial classes, including β-lactams, macrolides, and sulfonamides. The most frequently detected resistance genes were *msrA* (26.5%) and *mefA* (20.6%). All isolates formed biofilms, with 44.1% classified as strong producers and 55.9% as moderate producers. Strong biofilm formation was significantly associated with antimicrobial resistance (OR = 4.71, *p* = 0.045). **Conclusions:** *L. monocytogenes* was detected in 34 (2.18%) animal-origin food samples from Northern Kazakhstan. The isolates demonstrated antimicrobial resistance and biofilm-forming capacity, with a significant association between strong biofilm formation and antimicrobial resistance. These findings highlight the importance of continued microbiological surveillance of *L. monocytogenes* in animal-origin foods.

## 1. Introduction

*Listeria monocytogenes* is among the most important foodborne bacterial pathogens because of its ability to cause invasive listeriosis, a severe disease associated with high hospitalization rates. Mortality reaches 20–30% among invasive cases, particularly among pregnant women, newborns, older adults, and immunocompromised individuals [1,2]. The public health burden of this pathogen remains substantial: 2993 confirmed listeriosis cases were reported in the European Union in 2023, representing the highest annual number recorded since the establishment of EU-wide surveillance [3].

The ability of *L. monocytogenes* to persist throughout the food chain is closely linked to its exceptional physiological adaptability. This includes survival over a wide range of temperatures (0–45 °C), pH values (4.3–9.6), and salt concentrations (up to 10% NaCl), enabling the long-term contamination of foods and food-processing environments [4,5]. These traits have made *L. monocytogenes* a priority pathogen in food safety research and a key focus of the One Health approach, which promotes the integrated management of foodborne pathogens across the human, animal, and environmental sectors [6,7]. In addition to this environmental persistence, antimicrobial resistance (AMR) has become an increasing concern. Although β-lactam antibiotics, particularly ampicillin combined with aminoglycosides, remain the first-line treatment for invasive listeriosis [8], resistance to tetracyclines, macrolides, and fluoroquinolones has been reported worldwide, particularly among foodborne isolates [9,10,11]. At the molecular level, this resistance is associated with genes such as *tetM*, *tetS*, *ermB*, *lde*, and *mdrL*, which confer resistance through efflux and target-modification mechanisms. These genes are frequently carried on mobile genetic elements, including *Tn916*-like transposons and plasmids, which facilitate horizontal dissemination [12,13,14,15]. Combined with the extensive use of antimicrobials in food-producing animals, these mechanisms contribute to the emergence of multidrug-resistant (MDR) strains—defined as strains resistant to at least three antimicrobial classes—which may persist within the food chain and compromise treatment efficacy [16,17].

This resistance is further compounded by the biofilm-forming ability of *L. monocytogenes*. Animal-derived foods, particularly meat, fish, and dairy products, are major vehicles for transmission to humans and for food chain contamination [18]. The persistence of this pathogen in food-processing environments is largely sustained by biofilm formation on food-contact surfaces. Biofilms enhance bacterial tolerance to disinfectants and antimicrobial agents, promoting long-term survival and recurrent contamination [19,20]. Biofilm formation is influenced by environmental conditions and bacterial physiological adaptation, and its regulation involves efflux and stress-response mechanisms [21]. In *L. monocytogenes*, the stress-response regulator SigB and the virulence regulator PrfA have been shown to contribute to biofilm formation under specific environmental conditions [22,23].

The combined ability of *L. monocytogenes* to persist through biofilm formation and develop antimicrobial resistance highlights the importance of region-specific surveillance. Because the distribution, resistance patterns, and genetic characteristics of *L. monocytogenes* vary considerably across regions and food production systems, continuous regional surveillance is essential for understanding global epidemiology and strengthening international monitoring programs, such as the WHO Global Antimicrobial Resistance and Use Surveillance System (GLASS) [24]. In Kazakhstan, however, published studies have focused mainly on the microbiological safety of foods and the epizootiological surveillance of listeriosis, while the phenotypic resistance, resistance genes, and biofilm-forming capacity of foodborne *L. monocytogenes* isolates remain largely uncharacterized [25,26]. In contrast, comparable investigations have already been conducted in neighboring countries such as China and Iran [27,28,29,30], underscoring the need for similar evidence from Kazakhstan and the broader Central Asian region, where the relationship between AMR and biofilm formation in animal-derived foods remains poorly understood.

Therefore, this study aimed to determine the occurrence, phenotypic AMR, antimicrobial resistance genes (ARGs), and biofilm-forming capacity of *L. monocytogenes* isolated from foods of animal origin in Northern Kazakhstan and to assess the association between biofilm-forming capacity and phenotypic AMR.

## 2. Results

### 2.1. Occurrence of L. monocytogenes in Foods of Animal Origin

A total of 1561 food samples of animal origin were analyzed between January 2024 and February 2026. *L. monocytogenes* was isolated from 34 samples, corresponding to an overall positivity rate of 2.18% (34/1561) (Table 1).

The detection rate varied among food categories. *L. monocytogenes* was detected in 4/600 (0.67%) raw milk samples, 0/120 (0%) dairy product samples, 7/220 (3.18%) red meat samples, 2/66 (3.03%) frozen semifinished product samples, 0/67 (0%) processed meat product samples, 14/346 (4.05%) poultry meat samples, and 7/142 (4.93%) chicken egg samples. The highest detection rates were observed in chicken eggs (4.93%) and poultry meat (4.05%), whereas no isolates were recovered from dairy products or processed meat products.

### 2.2. Antimicrobial Susceptibility and Resistance Profiles of L. monocytogenes Isolates

**Figure 1:**
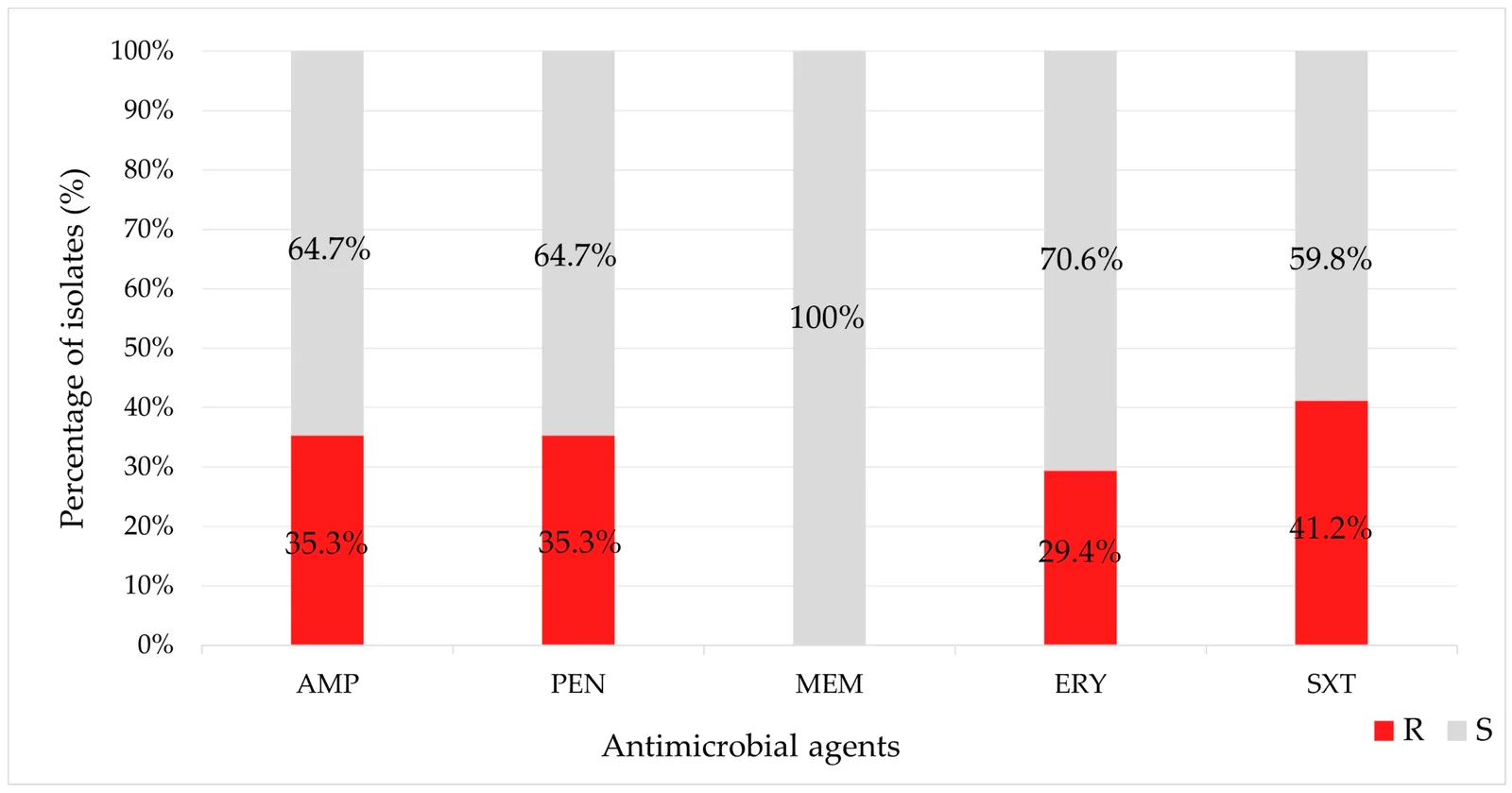
Distribution of susceptible and resistant *L. monocytogenes* isolates across the tested antimicrobial agents. AMP, ampicillin; PEN, benzylpenicillin; MEM, meropenem; ERY, erythromycin; SXT, trimethoprim/sulfamethoxazole. R, resistant isolates; S, susceptible isolates. The values are presented as percentages of the total number of isolates tested.

The antimicrobial susceptibility profiles of the 34 *L. monocytogenes* isolates are presented in Figure 1. All isolates were susceptible to meropenem (100%). Phenotypic resistance to at least one antimicrobial agent was detected in 52.9% (18/34) of the isolates, whereas 47.1% (16/34) were susceptible to all antibiotics tested.

The highest resistance rate was observed for trimethoprim/sulfamethoxazole, to which 41.2% (14/34) of the isolates were resistant. Resistance to ampicillin and benzylpenicillin was observed in 35.3% (12/34) of the isolates, with resistance to both agents present in 32.4% (11/34) of the isolates. Erythromycin resistance was identified in 29.4% (10/34) of the isolates.

Several phenotypic AMR profiles were identified (Table 2). The predominant profile was AMP–PEN–ERY–SXT, representing combined resistance to β-lactam antibiotics, macrolides, and trimethoprim/sulfamethoxazole, which was identified in 23.5% (8/34) of the isolates. This profile was found exclusively among isolates recovered from red meat (beef, horse meat, and mutton) and raw milk, where it accounted for 71.4% (5/7) and 75.0% (3/4) of the isolates, respectively. No isolates with this profile were recovered from poultry meat, chicken eggs, or frozen semifinished products.

Phenotypic AMR profiles varied according to the source of isolation. Among the poultry meat isolates, 50.0% (7/14) were susceptible to all antimicrobial agents tested. Among the resistant isolates, exclusive resistance to trimethoprim/sulfamethoxazole was the most common phenotype, accounting for 21.4% (3/14). Combined resistance phenotypes were observed in 28.6% (4/14) of the isolates and included the PEN–SXT, AMP–SXT, AMP–PEN–ERY, and AMP–PEN–SXT profiles.

MDR was detected in 8/34 (23.5%) isolates: LM13, LM15, LM16, LM17, LM18, LM28, LM29, and LM30. All MDR isolates were resistant to ampicillin, benzylpenicillin, erythromycin, and trimethoprim/sulfamethoxazole, corresponding to resistance to three antimicrobial classes: β-lactams, macrolides, and sulfonamides. Five MDR isolates were recovered from red meat and three from raw cow milk, whereas no MDR isolates were identified among poultry meat, chicken eggs, or frozen semifinished products.

### 2.3. ARGs in L. monocytogenes Isolates

Comparisons of genotypic and phenotypic resistance profiles revealed variable concordance among the resistance genes investigated (Table 3).

The remaining ARGs included in the PCR screening (*tetB*, *tetK*, *tetL*, *ermA*, *strA*, *aadA*, *sul1*, *sul2*, and *dfrD*) were not detected in any of the *L. monocytogenes* isolates. None of the *mefA*-positive isolates exhibited phenotypic resistance to erythromycin, whereas erythromycin resistance was observed in 7 of the 9 *msrA*-positive isolates and in the single *ermB*-positive isolate. The *tetA* and *tetM* genes were detected in isolates with different phenotypic resistance profiles for the antimicrobial agents tested in this study. Conversely, several isolates exhibiting phenotypic resistance to β-lactams, macrolides, or trimethoprim/sulfamethoxazole did not carry any of the ARGs investigated.

Among the MDR isolates, *msrA* was the predominant resistance gene, with six of the nine *msrA*-positive isolates (66.7%) exhibiting an MDR phenotype. These isolates originated predominantly from red meat and raw cow milk and were characterized by the combined resistance profiles AMP–PEN–ERY–SXT and AMP–PEN–ERY.

### 2.4. Biofilm-Forming Capacity of L. monocytogenes Isolates

All 34 *L. monocytogenes* isolates examined were capable of biofilm formation, with isolates distributed between strong and moderate biofilm-producing categories according to the classification proposed by Stepanović et al. [31] (Table 4). No weak or non-biofilm-producing isolates were identified.

**Figure 2:**
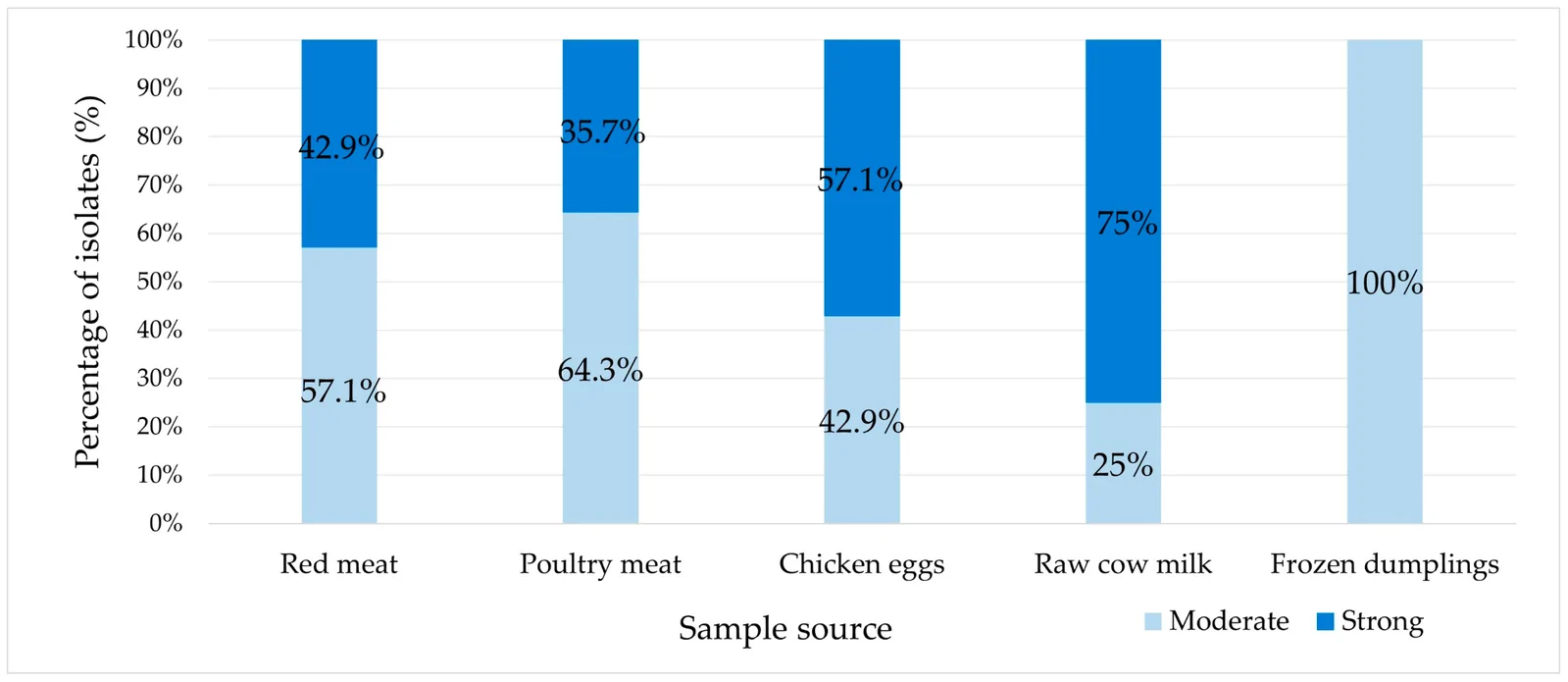
Distribution of biofilm-forming intensity among *L*. *monocytogenes* isolates according to the source of isolation.

The distribution of biofilm-forming intensity varied by source of isolation (Figure 2). Among the isolates from red meat (n = 7), 57.1% (4/7) were classified as moderate biofilm producers, whereas 42.9% (3/7) were strong biofilm producers. A similar pattern was observed among the isolates from poultry meat (n = 14), with 64.3% (9/14) classified as moderate and 35.7% (5/14) as strong biofilm producers. In contrast, isolates from raw cow milk were predominantly strong biofilm producers (75.0%; 3/4), whereas only 25.0% (1/4) were classified as moderate biofilm producers.

Among the isolates recovered from chicken eggs (n = 7), strong biofilm producers also predominated, accounting for 57.1% (4/7) of the isolates, whereas 42.9% (3/7) were classified as moderate biofilm producers. All isolates recovered from frozen dumplings (n = 2) were classified as moderate biofilm producers (100%; 2/2). Despite the observed differences in the distribution of biofilm-forming phenotypes, no statistically significant association was detected between the source of isolation and biofilm-forming intensity (Fisher’s exact test, *p* = 0.483).

### 2.5. Association Between AMR and the Biofilm-Forming Capacity of L. monocytogenes Isolates

A greater proportion of AMR isolates was observed among strong biofilm producers (73.3%; 11/15) than among moderate biofilm producers (36.8%; 7/19) (Table 5).

**Figure 3:**
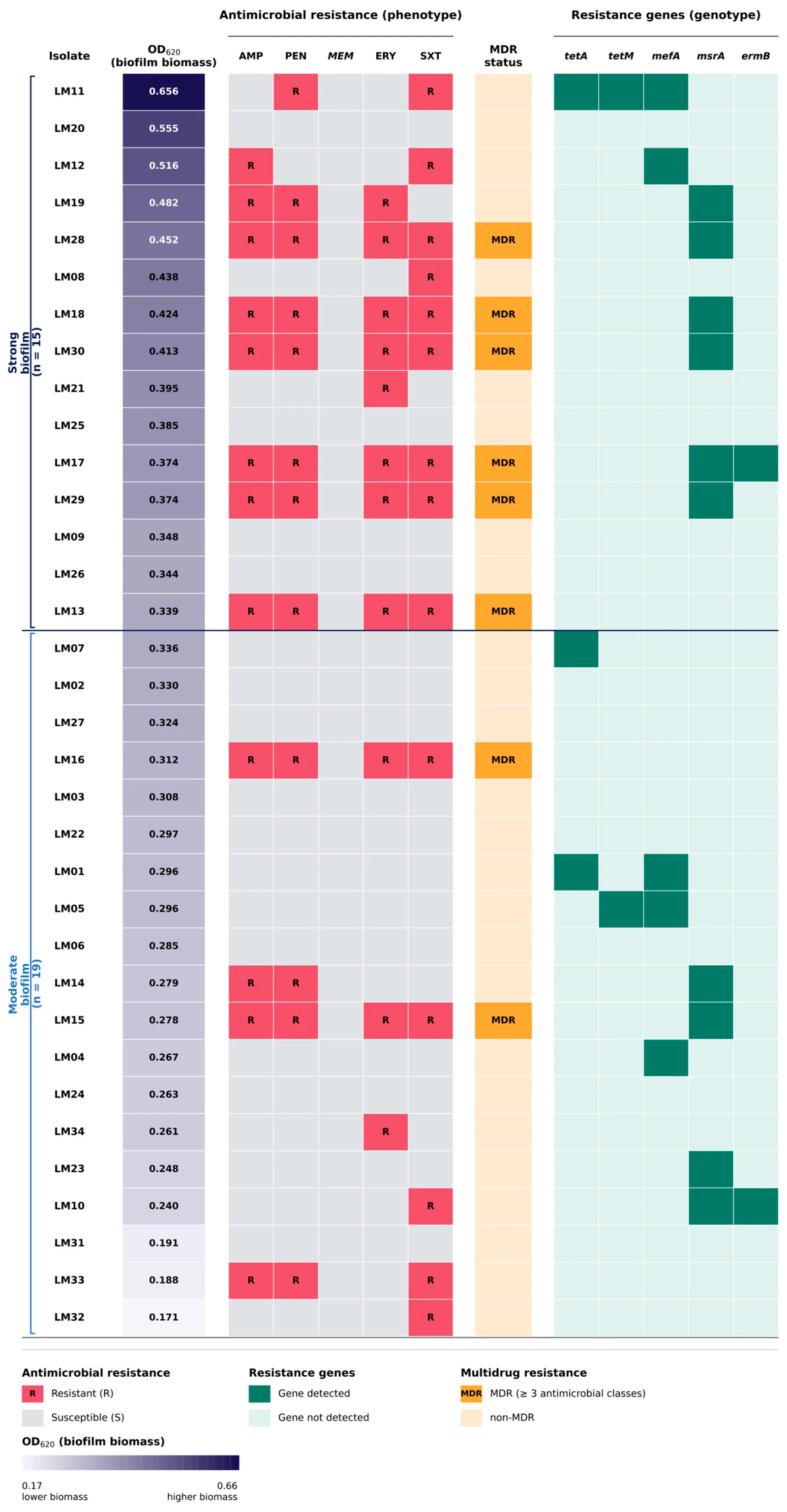
Integrated visualization of the relationships among biofilm-forming capacity, phenotypic AMR, and the distribution of ARGs in *L*. *monocytogenes* isolates. Isolates were ordered according to their biofilm biomass on the basis of their OD_620_ values. The left panel shows the biofilm phenotype (strong or moderate) and biofilm biomass (OD_620_). The middle panel presents the phenotypic susceptibility profiles for ampicillin (AMP), benzylpenicillin (PEN), meropenem (MEM), erythromycin (ERY), and trimethoprim/sulfamethoxazole (SXT), together with the MDR status. The right panel illustrates the distribution of the ARGs *tetA*, *tetM*, *mefA*, *msrA*, and *ermB* detected among the studied isolates.

Statistical analysis revealed a significant association between biofilm-forming capacity and AMR. Strong biofilm producers were 4.71 times more likely to exhibit an AMR phenotype than moderate biofilm producers were (OR = 4.71, 95% CI: 1.08–20.63; two-tailed Fisher’s exact test, *p* = 0.045). To further elucidate the relationships among the biofilm-forming capacity, phenotypic AMR, MDR status, and distribution of ARGs, a comparative analysis of all *L. monocytogenes* isolates was performed (Figure 3).

A higher proportion of AMR isolates was observed among strong biofilm producers, whereas phenotypically susceptible isolates predominated among moderate biofilm producers. Nevertheless, both AMR and susceptible isolates were represented in each biofilm category. ARGs were detected in both biofilm categories, with no apparent association between ARG distribution and biofilm-forming capacity. The efflux-associated genes *msrA* and *mefA* were the most frequently detected determinants, whereas *tetA*, *tetM*, and *ermB* occurred only sporadically. MDR phenotypes were observed in both strong and moderate biofilm producers.

## 3. Discussion

Although *L. monocytogenes* was detected at a relatively low frequency in the analyzed food products in the present study (2.18%; 34/1561), the phenotypic characteristics of the recovered isolates suggest a potential public health concern. The coexistence of AMR and biofilm-forming capacity may increase environmental persistence and facilitate survival during food processing, underscoring the importance of continuous monitoring and effective control strategies.

The occurrence of *L. monocytogenes* in poultry-derived products in the present study was 4.05% (14/346) in poultry meat and 4.93% (7/142) in chicken eggs. By comparison, reported positivity values in the literature are 8.32% in meat products in South Korea, 7.1% in livestock and poultry meat in China, and 8.3% in the EU [32,33], while the prevalence of *L. monocytogenes* on eggshells has been reported to range from 1.8% to 6.0% [34]. These findings align with the variability in *L. monocytogenes* contamination reported across food products in different countries [35]. Differences in contamination levels among poultry-derived products are likely influenced by production systems, hygienic conditions, food processing practices, and food safety management [36]. In Taiwan, *L. monocytogenes* was detected in 11.4% of postevisceration chicken carcass rinse samples, while 16.7% positivity was reported in raw materials in a Spanish pork-processing industry [37,38]. Intensive poultry production, cross-contamination during slaughter and processing, and the psychrotrophic nature of *L. monocytogenes*—which enables survival under refrigeration—may further contribute to its occurrence in poultry and other meat products [37,39,40]. Such differences may also be related to variations in food matrices, sampling strategies, processing stages, and the persistence of *L. monocytogenes* in food-processing environments [41].

Conversely, the occurrence of *L. monocytogenes* in raw cow’s milk in the present study was 0.67% (4/600), whereas no positive samples were detected among processed dairy products or sausages. This percentage was lower than the 7.69% positivity reported in raw milk samples from South Africa [42]. Global evidence also indicates substantial variation in *L. monocytogenes* occurrence across milk and dairy product supply chains [43]. The lower occurrence observed in the present study may be associated with differences in milking hygiene, environmental exposure at the farm level, and asymptomatic carriage among dairy animals [44]. These factors may contribute to the contamination of raw milk at the primary production stage, whereas subsequent processing and hygienic control may reduce the occurrence of *L. monocytogenes* in processed products [43,44].

Phenotypic antimicrobial susceptibility testing revealed that more than half of the *L. monocytogenes* isolates were resistant to at least one antimicrobial agent, indicating the presence of antimicrobial-resistant strains among the analyzed food isolates. All isolates remained susceptible to meropenem, which is consistent with the intrinsically high susceptibility of *L. monocytogenes* to carbapenems reported previously [45,46]. Resistance to trimethoprim/sulfamethoxazole was most frequently observed, followed by resistance to ampicillin, benzylpenicillin, and erythromycin. The predominance of resistance to these antimicrobial agents is consistent with findings reported for food-derived *L. monocytogenes* in other countries [47,48,49]. However, antimicrobial susceptibility profiles vary across regions, likely reflecting differences in antimicrobial use in food-producing animals, national antimicrobial stewardship policies, and the circulation of distinct *L. monocytogenes* lineages. Because ampicillin remains a first-line treatment for invasive listeriosis, the detection of ampicillin- and benzylpenicillin-resistant isolates raises concerns about clinically relevant resistance and supports continued surveillance of antimicrobial resistance in food-derived *L. monocytogenes* [50].

MDR was observed predominantly among isolates recovered from beef, horse meat, and raw cow’s milk, whereas poultry-derived isolates exhibited comparatively low MDR frequencies, which aligns with previous reports describing source-dependent differences in antimicrobial resistance among foodborne *L. monocytogenes* isolates [51,52]. These differences most likely reflect variations in antimicrobial exposure, animal husbandry practices, veterinary antimicrobial stewardship, surveillance programs, and the circulation of distinct *L. monocytogenes* lineages across production systems [53,54].

Molecular analysis revealed that nearly half of the *L. monocytogenes* isolates carried at least one antimicrobial resistance gene, with the efflux-associated genes *msrA* and *mefA* predominating. Similar distributions have been reported for food-derived *L. monocytogenes*, suggesting that antimicrobial efflux is a major adaptive mechanism enabling survival during antimicrobial exposure [45,55]. In contrast, genes associated with ribosomal protection and target modification were detected only sporadically, indicating that these mechanisms made a limited contribution to the resistance profiles observed in the present study.

The distribution of resistance determinants also varied among food sources. The *msrA* gene was more frequently detected in isolates from beef, horse meat, and raw cow’s milk. Although the limited number of isolates precludes definitive conclusions, this pattern may reflect differences in antimicrobial selection pressure and the circulation of distinct *L. monocytogenes* lineages across production systems [56].

The relationship between genotypic resistance and phenotypic resistance was not completely concordant. Several resistance genes were detected in phenotypically susceptible isolates, whereas some phenotypically resistant isolates lacked the resistance determinants included in the present screening panel. Similar discrepancies have been reported previously and may reflect differences in gene expression, regulatory mechanisms, chromosomal mutations, or resistance determinants not included in the current screening panel [45,57]. These findings indicate that phenotypic susceptibility testing and molecular characterization provide complementary information and should be interpreted together when AMR in *L. monocytogenes* is being assessed.

All *L. monocytogenes* isolates demonstrated the ability to form biofilms, with moderate and strong biofilm producers predominating. Similar findings have been widely reported for food-derived *L. monocytogenes*, indicating that biofilm formation is a conserved survival strategy enabling persistence under food-processing conditions. The extracellular polymeric matrix protects embedded cells from disinfectants and environmental stresses while promoting surface attachment and persistence on food-contact materials, thereby facilitating the recurrent contamination of processing environments [58,59]. These findings support the implementation of sanitation strategies specifically targeting biofilm-associated *L. monocytogenes* in food-processing facilities.

A significant association was observed between biofilm-forming capacity and phenotypic antimicrobial resistance, with strong biofilm producers being more likely to exhibit an AMR phenotype than moderate producers. Similar associations have been reported for *L. monocytogenes* and other foodborne pathogens, suggesting that enhanced biofilm formation and AMR may represent complementary adaptive responses that increase bacterial persistence under environmental and antimicrobial stress, rather than reflecting a direct causal relationship [60,61]. Although MDR occurred more frequently among strong biofilm producers, this association was not statistically significant, most likely because of the limited sample size. Similarly, no clear relationship was observed between individual resistance genes and biofilm-forming capacity, indicating that biofilm formation is regulated by complex networks involving stress responses, quorum sensing, virulence-associated factors, and global transcriptional regulators, rather than individual resistance determinants alone [58]. Similar observations have been reported previously, supporting the role of these regulatory networks in biofilm development [62].

Several limitations should be acknowledged. The relatively small number of isolates, limited geographical coverage, and targeted screening of a restricted set of antimicrobial resistance genes may have resulted in an underestimation of the diversity of resistance mechanisms among food-derived *L. monocytogenes*. In addition, sequence typing (ST) and serotyping were not performed in the present study, limiting the assessment of the molecular epidemiological diversity and serotype distribution of the isolates.

An additional limitation of the present study is that the virulence potential and clonal structure of the *L. monocytogenes* isolates were not investigated. Virulence in *L. monocytogenes* is a multifactorial trait, and the presence of individual virulence-associated genes alone does not allow reliable classification of isolates according to their virulence level. Current approaches distinguish several hypervirulence-associated clonal complexes, including CC1, CC2, CC4, and CC6, from predominantly food-associated hypovirulent clones such as CC9 and CC121 on the basis of population-genetic, epidemiological, genomic, and experimental evidence. Other geographically relevant clones, including the hypervirulence-associated CC87 reported particularly in Asia, further illustrate the diversity of virulence-associated lineages. Comprehensive characterization of the present isolate collection would therefore require molecular typing to determine STs/CCs together with analysis of virulence-associated genomic determinants, including LIPI-1, LIPI-3, LIPI-4, internalin genes, and *inlA* integrity; WGS would provide the most comprehensive framework for such an analysis. Importantly, genomic virulence profiles represent predictions of virulence potential and do not necessarily correspond directly to functional virulence, which may require complementary cell-based or in vivo assays. Such integrated characterization was beyond the predefined objectives of the present study, which focused on antimicrobial resistance, resistance-associated genes, biofilm-forming capacity, and their relationship. Nevertheless, we consider comprehensive characterization of virulence potential and population structure an important next step and plan to investigate these characteristics in this *L. monocytogenes* collection in a dedicated follow-up study.

Future studies should include larger isolate collections from broader geographical areas and incorporate serotyping, multilocus sequence typing (MLST), and whole-genome sequencing to provide a more comprehensive characterization of antimicrobial resistance, virulence determinants, molecular epidemiology, and persistence-associated traits, including resistance to disinfectants.

## 4. Materials and Methods

### 4.1. Study Design

This study was designed as a descriptive observational study to characterize *L. monocytogenes* isolates recovered from animal-derived food products in the Kostanay Region, Northern Kazakhstan. The study was conducted from January 2024 to February 2026 and included samples collected at different stages of the food supply chain, including livestock farms, wholesale distribution centers, and retail outlets. This study involved the collection of animal-derived food products, the isolation and identification of *L. monocytogenes,* and the subsequent assessment of antimicrobial susceptibility, biofilm-forming capacity, and resistance-associated genetic determinants.

### 4.2. Sample Collection

The *L. monocytogenes* isolates included in this study were recovered between January 2024 and February 2026 in the Kostanay Region, Kazakhstan. The isolates were obtained from animal-derived food products, including both raw materials and ready-to-eat products, collected at different stages of the food supply chain, including farms, wholesale distribution centers, and retail outlets.

A total of 1561 samples were examined, comprising 600 samples of raw cow’s milk, 120 dairy products, 220 meat samples, 66 frozen semifinished products, 67 sausage products, 346 poultry meat samples, and 142 chicken egg samples.

Sample collection, transportation, and storage were performed according to ISO/TS 17728:2015 [63]. Samples were collected aseptically in sterile containers, transported at 4 ± 2 °C, and delivered to the laboratory within 24 h of collection.

### 4.3. Isolation of L. monocytogenes

*L. monocytogenes* was isolated from animal-derived food products using a culture-based method according to ISO 11290-1:2017 [64].

Primary and secondary enrichment were performed in Half Fraser Broth and Fraser Broth (Condalab, Madrid, Spain), with sequential incubation at 30 ± 1 °C for 24 ± 2 h, followed by incubation at 37 ± 1 °C for an additional 24 ± 2 h.

Following enrichment, culture aliquots were streaked onto CHROMagar™ Listeria and CHROMagar™ Identification Listeria (CHROMagar, Paris, France) and incubated at 37 ± 1 °C for 24–48 h. Presumptive *L. monocytogenes* colonies appeared blue on CHROMagar™ Listeria and pink with an opaque halo on CHROMagar™ Identification Listeria.

### 4.4. Identification of L. monocytogenes

Final species identification was performed using MALDI–TOF MS on a MALDI Biotyper Sirius System IVD (Bruker Daltonics GmbH & Co. KG, Bremen, Germany). The acquired mass spectra were compared against the manufacturer’s reference database, and isolates with a log(score) value ≥ 2.0 were considered reliably identified at the species level.

### 4.5. Antimicrobial Susceptibility Testing

The antimicrobial susceptibility of *L. monocytogenes* isolates was determined using the Kirby–Bauer disk diffusion method on Mueller–Hinton agar (HiMedia Laboratories Pvt. Ltd., Mumbai, India) supplemented with 5% defibrinated horse blood and β-NAD (Servicebio, Wuhan, China; 20 mg/L), in accordance with the recommendations of the EUCAST [65].

The bacterial inoculum was adjusted to a 0.5 McFarland standard (approximately 1.5 × 10^8^ CFU/mL) by measuring the turbidity of the bacterial suspension using a DEN-1 densitometer (SIA BIOSAN, Riga, Latvia) and adjusting the suspension until the 0.5 McFarland value was reached. Following the inoculation and placement of antimicrobial disks (HiMedia Laboratories Pvt. Ltd., Mumbai, India), the plates were incubated at 35–37 °C for 18–24 h, after which the diameters of the growth inhibition zones were measured (mm).

The antimicrobial panel included ampicillin, benzylpenicillin, meropenem, erythromycin, and trimethoprim/sulfamethoxazole. These agents were selected because EUCAST version 16.0 provides applicable disk diffusion breakpoints for their interpretation in *L. monocytogenes*. The susceptibility results were interpreted as susceptible (S) or resistant (R) according to the EUCAST breakpoints [65] using the zone diameter breakpoints provided in Table 6.

MDR was defined according to the international criteria proposed by Magiorakos et al. [66]. Isolates were classified as MDR when they exhibited resistance to at least one antimicrobial agent in three or more antimicrobial classes.

### 4.6. PCR Detection of ARGs

Genomic DNA was extracted using the AmpliSens DNA-sorb-B DNA Extraction Kit (Central Research Institute of Epidemiology, Moscow, Russia) according to the manufacturer’s instructions. DNA concentration and purity were determined spectrophotometrically using a NanoDrop One spectrophotometer (Thermo Fisher Scientific, Waltham, MA, USA). The DNA samples were stored at −20 °C until further analysis.

To identify antimicrobial resistance (AMR) determinants in *L. monocytogenes*, real-time PCR assays were performed to detect genes associated with resistance to tetracyclines (*tetA*, *tetB*, *tetK*, *tetL*, and *tetM*), macrolides (*ermA*, *ermB*, *mefA*, and *msrA*), aminoglycosides (*strA* and *aadA*), sulfonamides (*sul1* and *sul2*), and trimethoprim (*dfrD*).

Genomic DNA was extracted using the AmpliSens DNA-sorb-B DNA Extraction Kit (Central Research Institute of Epidemiology, Moscow, Russia) according to the manufacturer’s instructions. Real-time PCR amplification was performed using a QuantStudio™ 5 Real-Time PCR System (Applied Biosystems, Thermo Fisher Scientific, Waltham, MA, USA). Each 25 μL reaction mixture consisted of 12.5 μL of BioMaster UDG HS-qPCR Lo-ROX SYBR (2×) (Biolabmix, Novosibirsk, Russia), 2.5 μL each of the forward and reverse primers (10 pmol each; National Center for Biotechnology, Astana, Kazakhstan), 5 μL of template DNA, and 2.5 μL of nuclease-free water. The PCR cycling conditions were as follows: 50 °C for 2 min, 95 °C for 5 min, followed by 40 cycles of 95 °C for 10 s, annealing at the gene-specific temperature (Table 7) for 10 s, and 72 °C for 20 s. A melting curve analysis (65–95 °C) was performed after amplification to verify product specificity.

### 4.7. Assessment of Biofilm-Forming Capacity

The biofilm-forming capacity of *L. monocytogenes* isolates was assessed using a crystal violet microtiter plate assay. A bacterial suspension standardized to 0.5 McFarland (approximately 1.5 × 10^8^ CFU/mL) was diluted 1:100 and inoculated into sterile 96-well polystyrene microtiter plates containing tryptic soy broth (TSB; Condalab, Madrid, Spain) supplemented with 1% glucose. The plates were incubated at 37 °C for 24 h. Sterile TSB without bacterial inoculum was used as the negative control.

Following incubation, the wells were gently washed with phosphate-buffered saline (PBS; HiMedia Laboratories Pvt. Ltd., Mumbai, India) to remove nonadherent cells. The remaining attached biofilms were fixed and stained with 0.1% crystal violet solution, after which the bound dye was eluted with 33% acetic acid. The optical density (OD) was measured at 620 nm using a Multiskan microplate reader (Thermo Fisher Scientific, Waltham, MA, USA).

All experiments were performed in triplicate. The biofilm-forming capacity of the isolates was classified according to the criteria proposed by Stepanović et al. [31]. The optical density cutoff value (ODc) was calculated using the following formula:

(1) $$\mathrm{ODc}\;=\;{\bar{\mathrm{OD}}}_{\mathrm{negative}\;\mathrm{control}}\;+\;(3\;\times \;SD_{\mathrm{negative}\;\mathrm{control}}),$$

where ${\bar{\mathrm{OD}}}_{\mathrm{negative}\;\mathrm{control}}$—represents the mean optical density of the negative control and $SD_{\mathrm{negative}\;\mathrm{control}}$—represents the corresponding standard deviation.

Isolates were classified as nonbiofilm producers (OD ≤ ODc), weak biofilm producers (ODc < OD ≤ 2 × ODc), moderate biofilm producers (2 × ODc < OD ≤ 4 × ODc), or strong biofilm producers (OD > 4 × ODc).

### 4.8. Statistical Analysis

Statistical analyses were performed using jamovi software version 2.7.30 (The jamovi Project, Sydney, Australia). Categorical variables are expressed as absolute numbers (n) and percentages (%), whereas optical density (OD_620_) values are presented as the mean ± standard deviation (mean ± SD). Fisher’s exact test was used to compare categorical variables. The association between biofilm-forming capacity and the AMR phenotype was evaluated by calculating odds ratios (ORs) with corresponding 95% confidence intervals (95% CIs). Differences were considered to be statistically significant at *p* < 0.05.

## 5. Conclusions

This study provides evidence linking AMR and biofilm-forming capacity among foodborne *L. monocytogenes* isolates recovered from animal-derived foods in Northern Kazakhstan. The findings demonstrate that antimicrobial-resistant isolates with enhanced biofilm-forming capacity were present among the analyzed food samples, highlighting the relevance of these characteristics for food safety. The observed association between AMR and biofilm-forming capacity supports the importance of considering both phenotypic resistance and biofilm-forming ability in the microbiological monitoring of *L. monocytogenes* in animal-derived foods. These findings contribute to the understanding of AMR and the biofilm-associated characteristics of *L. monocytogenes* in food products from Northern Kazakhstan and emphasize the importance of integrated microbiological surveillance for food safety.

## Figures and Tables

**Table 1:** Distribution of *L. monocytogenes* isolates by source of isolation.

Source of Isolation	No. of Samples	No. of Isolates	Isolation Rate (%)
Raw milk (cow and mare)	600	4	0.67%
Dairy products (sour cream, butter, cheese, cottage cheese, etc.)	120	0	0%
Red meat (pork, beef, horse meat, mutton, and minced meat)	220	7	3.18%
Frozen semifinished products (dumplings, cutlets, etc.)	66	2	3.03%
Processed meat products (sausages, frankfurters, etc.)	67	0	0%
Poultry meat (chicken, turkey, and duck)	346	14	4.05%
Chicken eggs	142	7	4.93%
Total	1561	34	2.18%

**Table 2:** Phenotypic AMR profiles of *L. monocytogenes* isolates according to the source of isolation.

Isolate ID	Source	Resistance Patterns
LM01	Frozen dumplings	ND
LM02	Broiler chicken carcass	ND
LM03	Broiler chicken carcass	ND
LM04	Broiler chicken drumstick	ND
LM05	Broiler chicken breast	ND
LM06	Frozen dumplings	ND
LM07	Ground turkey	ND
LM08	Broiler chicken fillet	SXT
LM09	Broiler chicken fillet	ND
LM10	Broiler chicken breast	SXT
LM11	Broiler chicken fillet	PEN–SXT
LM12	Broiler chicken breast	AMP–SXT
LM13	Horse meat	AMP–PEN–ERY–SXT
LM14	Horse meat	AMP–PEN
LM15	Horse meat	AMP–PEN–ERY–SXT
LM16	Beef	AMP–PEN–ERY–SXT
LM17	Beef	AMP–PEN–ERY–SXT
LM18	Beef	AMP–PEN–ERY–SXT
LM19	Broiler chicken drumstick	AMP–PEN–ERY
LM20	Chicken egg	ND
LM21	Chicken egg	ERY
LM22	Chicken egg	ND
LM23	Chicken egg	ND
LM24	Chicken egg	ND
LM25	Chicken egg	ND
LM26	Chicken egg	ND
LM27	Raw cow milk	ND
LM28	Raw cow milk	AMP–PEN–ERY–SXT
LM29	Raw cow milk	AMP–PEN–ERY–SXT
LM30	Raw cow milk	AMP–PEN–ERY–SXT
LM31	Broiler chicken fillet	ND
LM32	Broiler chicken fillet	SXT
LM33	Broiler chicken drumstick	AMP–PEN–SXT
LM34	Mutton	ERY

Notes: LM01–LM34 represent individual *L. monocytogenes* isolates. The resistance patterns indicate the antimicrobial agents to which each isolate was resistant. AMP, ampicillin; PEN, benzylpenicillin; ERY, erythromycin; SXT, trimethoprim/sulfamethoxazole; ND, no resistance detected.

**Table 3:** Genotypic–phenotypic resistance profiles and sources of *L. monocytogenes* isolates.

Resistance Gene	Gene Class	Gene-Positive Isolates, *n*	Source of Isolation, *n*	Phenotypic Resistance Profile
*tetA*	Tetracycline	3	Poultry meat (2); frozen dumplings (1)	S (2); PEN–SXT (1)
*tetM*	Tetracycline	2	Poultry meat (2)	S (1); PEN–SXT (1)
*mefA*	Macrolide	7	Poultry meat (5); frozen dumplings (1); chicken eggs (1)	S (4); SXT (1); PEN–SXT (1); AMP–SXT (1)
*msrA*	Macrolide	9	Poultry meat (2); red meat (4); raw cow’s milk (3)	SXT (1); AMP–PEN (1); AMP–PEN–ERY–SXT (6); AMP–PEN–ERY (1)
*ermB*	Macrolide	1	Red meat (1)	AMP–PEN–ERY–SXT (1)

Notes: Values indicate the number of gene-positive isolates with the indicated phenotypic resistance profile. S, susceptible to all tested antibiotics; AMP, ampicillin; PEN, benzylpenicillin; ERY, erythromycin; SXT, trimethoprim/sulfamethoxazole.

**Table 4:** Distribution of *L. monocytogenes* isolates according to biofilm-forming ability.

Biofilm-Forming Category	No. of Isolates (*n*)	Percentage (%)	Mean OD_620_ ± SD
Strong biofilm producers	15	44.1	0.433 ± 0.088
Moderate biofilm producers	19	55.9	0.272 ± 0.048
Weak biofilm producers	0	0.0	ND
Nonbiofilm producers	0	0.0	ND

Notes: Biofilm-forming ability was classified as follows: OD ≤ ODc, no biofilm formation; ODc < OD ≤ 2ODc, weak biofilm formation; 2ODc < OD ≤ 4ODc, moderate biofilm formation; OD > 4ODc, strong biofilm formation. The cutoff optical density was ODc = 0.084. The OD_620_ values are presented as the mean ± SD. ND, not detected.

**Table 5:** Association between AMR and biofilm-forming ability in *L. monocytogenes* isolates.

Biofilm Category	AMR, *n* (%)	Non-AMR, *n* (%)	Total	OR (95% CI)	*p*-Value
Strong	11 (73.3)	4 (26.7)	15	4.71 (1.08–20.63)	0.045 *
Moderate	7 (36.8)	12 (63.2)	19	–	–
Total	18 (52.9)	16 (47.1)	34	–	–

Notes: AMR, antimicrobial resistance; OR, odds ratio; CI, confidence interval. * statistically significant at *p* < 0.05 (Fisher’s exact test).

**Table 6:** EUCAST zone diameter breakpoints used for antimicrobial susceptibility interpretation of *L. monocytogenes*.

Antibiotic	Disk Content	S (≥mm)	R (<mm)
Benzylpenicillin	1 IU	13	13
Ampicillin	2 μg	16	16
Meropenem	10 μg	26	26
Erythromycin	15 μg	25	25
Trimethoprim/sulfamethoxazole	1.25/23.75 μg	29	29

**Table 7:** Primers used for PCR detection of ARGs in *L. monocytogenes* isolates.

Gene	Primer Sequence (5′–3′)	Amplicon Size (bp)	Annealing Temperature (°C)	Reference
*tetA*	F: GCTACATCCTGCTTGCCTTC R: CATAGATCGCCGTGAAGAGG	210	55	[67]
*tetB*	F: TTGGTTAGGGGCAAGTTTTG R: GTAATGGGCCAATAACACCG	659	55	[67]
*tetK*	F: TCGATAGGAACAGCAGTA R: CAGCAGATCCTACTCCTT	169	55	[67]
*tetL*	F: TCGTTAGCGTGCTGTCATTC R: GTATCCCACCAATGTAGCCG	267	55	[67]
*tetM*	F: GTGGACAAAGGTACAACGAG R: CGGTAAAGTTCGTCACACAC	406	55	[67]
*ermA*	F: CTTCGATAGTTTATTAATATTAGT R: TCTAAAAAGCATGTAAAAGAA	645	52	[68]
*ermB*	F: GAAAAGGTACTCAACCAAATA R:AGTAACGGTACTTAAATTGTTTAC	636	52	[68]
*mefA*	F: AGTATCATTAATCACTAGTGC R: TTCTTCTGGTACTAAAAGTGG	345	52	[67]
*msrA*	F:GCAAATGGTGTAGGTAAGACAACT R: ATCATGTGATGTAAACAAAAT	401	52	[68]
*dfrD*	F: AGAGTAATCGGCAAGGATAACG R: AATGGGCAATTTCACAATCC	199	52	[68]
*strA*	F: CTTGGTGATAACGGCAATTC R: CCAATCGCAGATAGAAGGC	348	50	[67]
*aadA*	F: GTGGATGGCGGCCTGAAGCC R: AATGCCCAGTCGGCAGCG	525	50	[67]
*sul1*	F: CGGCGTGGGCTACCTGAACG R: GCCGATCGCGTGAAGTTCCG	433	65	[67]
*sul2*	F: GCGCTCAAGGCAGATGGCATT R: GCGTTTGATACCGGCACCCGT	293	65	[67]

## Data Availability

The data presented in this study are available from the corresponding author upon reasonable request.

## Abbreviations

AMR	Antimicrobial Resistance
ARG	Antimicrobial Resistance Gene
EUCAST	European Committee on Antimicrobial Susceptibility Testing
MDR	Multidrug Resistance

## References

[1] Osek J., Wieczorek K. (2022). *Listeria monocytogenes*—How This Pathogen Uses Its Virulence Mechanisms to Infect the Hosts. Pathogens.

[2] World Health Organization (2018). Listeriosis.

[3] European Centre for Disease Prevention and Control (2025). Listeriosis—Annual Epidemiological Report for 2023.

[4] Wiktorczyk-Kapischke N., Skowron K., Grudlewska-Buda K., Wałecka-Zacharska E., Korkus J., Gospodarek-Komkowska E. (2021). Adaptive Response of *Listeria monocytogenes* to the Stress Factors in the Food Processing Environment. Front. Microbiol..

[5] Hong H., Choi J., Kim H.J., Park S.H. (2024). Stress Resistance Insights of 65 *Listeria* Strains: Acidic, Low Temperature, and High Salt Environments. Microb. Pathog..

[6] Disson O., Moura A., Lecuit M. (2021). Making Sense of the Biodiversity and Virulence of *Listeria monocytogenes*. Trends Microbiol..

[7] Ryzhova E., Janine W., Chantelle H.D., Holý O. (2025). *Listeria monocytogenes* in Organic and Conventional Farming: Epidemiology, Risks, and Solutions within a One Health Framework. One Health.

[8] Kraus V., Čižmárová B., Birková A. (2024). *Listeria* in Pregnancy-The Forgotten Culprit. Microorganisms.

[9] Silva A., Silva V., Gomes J.P., Coelho A., Batista R., Saraiva C., Esteves A., Martins Â., Contente D., Diaz-Formoso L. (2024). *Listeria monocytogenes* from Food Products and Food Associated Environments: Antimicrobial Resistance, Genetic Clustering and Biofilm Insights. Antibiotics.

[10] Wilson A., Gray J., Chandry P.S., Fox E.M. (2018). Phenotypic and Genotypic Analysis of Antimicrobial Resistance among *Listeria monocytogenes* Isolated from Australian Food Production Chains. Genes.

[11] Ji S., Song Z., Luo L., Wang Y., Li L., Mao P., Ye C., Wang Y. (2023). Whole-Genome Sequencing Reveals Genomic Characterization of *Listeria monocytogenes* from Food in China. Front. Microbiol..

[12] Haubert L., Cruxen C.E.S., Fiorentini Â.M., da Silva W.P. (2018). Tetracycline Resistance Transfer from Foodborne *Listeria monocytogenes* to Enterococcus faecalis in Minas Frescal Cheese. Int. Dairy J..

[13] Lee M., Shin J.I., Hassan A., Chung Y.J., Jung S.H., Park K.T. (2026). Prevalence, Antimicrobial Resistance, and Genetic Characterization of *Listeria monocytogenes* in the Korean Pork Production Chain. Int. J. Food Microbiol..

[14] Jiang X., Yu T., Xu P., Xu X., Ji S., Gao W., Shi L. (2018). Role of Efflux Pumps in the In Vitro Development of Ciprofloxacin Resistance in *Listeria monocytogenes*. Front. Microbiol..

[15] Bertsch D., Uruty A., Anderegg J., Lacroix C., Perreten V., Meile L. (2013). Tn6198, a Novel Transposon Containing the Trimethoprim Resistance Gene dfrG Embedded into a Tn916 Element in *Listeria monocytogenes*. J. Antimicrob. Chemother..

[16] Wiśniewski P., Adamski P., Trymers M., Chajęcka-Wierzchowska W., Zadernowska A. (2025). Predicting Antibiotic Resistance in *Listeria monocytogenes* from Food and Food-Processing Environments Using Next-Generation Sequencing: A Systematic Review. Int. J. Mol. Sci..

[17] Manyi-Loh C.E., Lues R. (2025). *Listeria monocytogenes* and Listeriosis: The Global Enigma. Foods.

[18] Ricci A., Allende A., Bolton D., Chemaly M., Davies R., Fernández Escámez P.S., Girones R., Herman L., Koutsoumanis K., EFSA Panel on Biological Hazards (BIOHAZ) (2018). *Listeria monocytogenes* Contamination of Ready-to-Eat Foods and the Risk for Human Health in the EU. EFSA J..

[19] Vázquez-Sánchez D., Galvão J.A., Oetterer M. (2017). Contamination Sources, Serogroups, Biofilm-Forming Ability and Biocide Resistance of *Listeria monocytogenes* Persistent in Tilapia-Processing Facilities. J. Food Sci. Technol..

[20] Skowron K., Wałecka-Zacharska E., Grudlewska K., Gajewski P., Wiktorczyk N., Wietlicka-Piszcz M., Dudek A., Skowron K.J., Gospodarek-Komkowska E. (2019). Disinfectant Susceptibility of Biofilm Formed by *Listeria monocytogenes* under Selected Environmental Conditions. Microorganisms.

[21] Hall C.W., Mah T.F. (2017). Molecular Mechanisms of Biofilm-Based Antibiotic Resistance and Tolerance in Pathogenic Bacteria. FEMS Microbiol. Rev..

[22] van der Veen S., Abee T. (2010). Importance of SigB for *Listeria monocytogenes* Static and Continuous-Flow Biofilm Formation and Disinfectant Resistance. Appl. Environ. Microbiol..

[23] Lemon K.P., Freitag N.E., Kolter R. (2010). The Virulence Regulator PrfA Promotes Biofilm Formation by *Listeria monocytogenes*. J. Bacteriol..

[24] World Health Organization (2025). Global Antibiotic Resistance Surveillance Report 2025: WHO Global Antimicrobial Resistance and Use Surveillance System (GLASS).

[25] Kirimbayeva Z., Abutalip A., Mussayeva A., Kuzembekova G., Yegorova N. (2023). Epizootological Monitoring of Some Bacterial Infectious Diseases of Animals on the Territory of the Republic of Kazakhstan. Comp. Immunol. Microbiol. Infect. Dis..

[26] Bizhanov A., Adilov A., Yegorova N., Mussayeva A., Utegenova M., Kanatbaev S., Keikiyeva K., Sholpankulova A., Kanatov B. (2025). Listeriosis of Farm Animals and Measures of Struggle. Sci. Educ..

[27] Shen J., Zhang G., Yang J., Zhao L., Jiang Y., Guo D., Wang X., Zhi S., Xu X., Dong Q. (2022). Prevalence, Antibiotic Resistance, and Molecular Epidemiology of *Listeria monocytogenes* Isolated from Imported Foods in China during 2018–2020. Int. J. Food Microbiol..

[28] Mirzaei R., Golestan L., Fekrirad Z. (2024). *Listeria monocytogenes* Isolated from Ready-to-Eat Food Products in Tehran: Prevalence and Antimicrobial Resistance. Arch. Razi Inst..

[29] Farhoumand P., Hassanzadazar H., Soltanpour M.S., Aminzare M., Abbasi Z. (2020). Prevalence, Genotyping and Antibiotic Resistance of *Listeria monocytogenes* and Escherichia coli in Fresh Beef and Chicken Meats Marketed in Zanjan, Iran. Iran. J. Microbiol..

[30] Gao B., Cai H., Xu B., Yang F., Dou X., Dong Q., Yan H., Bu X., Li Z. (2024). Growth, Biofilm Formation, and Motility of *Listeria monocytogenes* Strains Isolated from Food and Clinical Samples Located in Shanghai, China. Food Res. Int..

[31] Stepanović S., Vuković D., Hola V., Di Bonaventura G., Djukić S., Cirković I., Ruzicka F. (2007). Quantification of Biofilm in Microtiter Plates: Overview of Testing Conditions and Practical Recommendations for Assessment of Biofilm Production by Staphylococci. APMIS.

[32] Je H.J., Kim U.I., Koo O.K. (2024). A Comprehensive Systematic Review and Meta-Analysis of *Listeria monocytogenes* Prevalence in Food Products in South Korea. Int. J. Food Microbiol..

[33] Zhang H., Luo X., Aspridou Z., Misiou O., Dong P., Zhang Y. (2023). The Prevalence and Antibiotic Resistance of *Listeria monocytogenes* in Livestock and Poultry Meat in China and the EU from 2001 to 2022: A Systematic Review and Meta-Analysis. Foods.

[34] Ricke S.C., O’Bryan C.A., Rothrock M.J. (2023). *Listeria* Occurrence in Conventional and Alternative Egg Production Systems. Microorganisms.

[35] Hamidiyan N., Salehi-Abargouei A., Rezaei Z., Dehghani-Tafti R., Akrami-Mohajeri F. (2018). The Prevalence of *Listeria* spp. Food Contamination in Iran: A Systematic Review and Meta-Analysis. Food Res. Int..

[36] Khan I., Khan J., Miskeen S., Tango C.N., Park Y.S., Oh D.H. (2016). Prevalence and control of *Listeria monocytogenes* in the food industry—A review. Czech J. Food Sci..

[37] Lin C.H., Adams P.J., Huang J.F., Sun Y.F., Lin J.H., Robertson I.D. (2021). Contamination of Chicken Carcasses and the Abattoir Environment with *Listeria monocytogenes* in Taiwan. Br. Poult. Sci..

[38] Romero de Castilla López B., Gómez Lozano D., Herrera Marteache A., Conchello Moreno P., Rota García C. (2025). Control of Persistent *Listeria monocytogenes* in the Meat Industry: From Detection to Prevention. Foods.

[39] Cavalcanti A.A.C., Limeira C.H., Siqueira I.N., Lima A.C., Medeiros F.J.P., Souza J.G., Medeiros N.G.A., Oliveira Filho A.A., Melo M.A. (2022). The Prevalence of *Listeria monocytogenes* in Meat Products in Brazil: A Systematic Literature Review and Meta-Analysis. Res. Vet. Sci..

[40] Rothrock M.J., Micciche A.C., Bodie A.R., Ricke S.C. (2019). *Listeria* Occurrence and Potential Control Strategies in Alternative and Conventional Poultry Processing and Retail. Front. Sustain. Food Syst..

[41] Tuytschaever T., Raes K., Sampers I. (2023). *Listeria monocytogenes* in Food Businesses: From Persistence Strategies to Intervention/Prevention Strategies-A Review. Compr. Rev. Food Sci. Food Saf..

[42] Kayode A.J., Okoh A.I. (2022). Assessment of Multidrug-Resistant *Listeria monocytogenes* in Milk and Milk Products from a One Health Perspective. PLoS ONE.

[43] Li X., Zheng J., Zhao W., Wu Y. (2024). Prevalence of *Listeria monocytogenes* in Milk and Dairy Product Supply Chains: A Global Systematic Review and Meta-Analysis. Foodborne Pathog. Dis..

[44] Rodríguez C., Taminiau B., Delmée M., Daube G. (2021). *Listeria monocytogenes* Dissemination in Farming and Primary Production: Sources, Shedding and Control Measures. Food Control.

[45] Wiśniewski P., Zakrzewski A.J., Zadernowska A., Chajęcka-Wierzchowska W. (2022). Antimicrobial Resistance and Virulence Characterization of *Listeria monocytogenes* Strains Isolated from Food and Food Processing Environments. Pathogens.

[46] Liu Y., Zhang P., Wang D., Wang B., Zhang Y., Zhang X. (2025). Prevalence and Characteristics of *Listeria monocytogenes* in Ready-to-Eat Chilled Pot Skewer Products. Front. Microbiol..

[47] Skowron K., Wałecka-Zacharska E., Wiktorczyk-Kapischke N., Skowron K.J., Grudlewska-Buda K., Bauza-Kaszewska J., Bernaciak Z., Borkowski M., Gospodarek-Komkowska E. (2020). Assessment of the Prevalence and Drug Susceptibility of *Listeria monocytogenes* Strains Isolated from Various Types of Meat. Foods.

[48] Paiva J., Silva V., Poeta P., Saraiva C. (2025). Antimicrobial Resistance Profile and Biofilm Formation of *Listeria monocytogenes* Isolated from Meat. Antibiotics.

[49] Elsayed M.E., Abd El-Hamid M.I., El-Gedawy A., Bendary M.M., ELTarabili R.M., Alhomrani M., Alamri A.S., Alghamdi S.A., Arnout M., Binjawhar D.N. (2022). New Insights into Listeria monocytogenes Antimicrobial Resistance, Virulence Attributes and Their Prospective Correlation. Antibiotics.

[50] Dos Reis J.O., Vieira B.S., Cunha Neto A., Silva M.C., Hoffmann C., Fritzen J.T.T., Steffens C., Furian T.Q., Borges K.A., Salle C.T.P. (2022). Antimicrobial Resistance of *Listeria monocytogenes* from Animal Foods to First- and Second-Line Drugs in the Treatment of Listeriosis from 2008 to 2021: A Systematic Review and Meta-Analysis. Can. J. Infect. Dis. Med. Microbiol..

[51] Matheou A., Abousetta A., Pascoe A.P., Papakostopoulos D., Charalambous L., Panagi S., Panagiotou S., Yiallouris A., Filippou C., Johnson E.O. (2025). Antibiotic Use in Livestock Farming: A Driver of Multidrug Resistance?. Microorganisms.

[52] Rippa A., Bilei S., Peruzy M.F., Marrocco M.G., Leggeri P., Bossù T., Murru N. (2024). Antimicrobial Resistance of *Listeria monocytogenes* Strains Isolated in Food and Food-Processing Environments in Italy. Antibiotics.

[53] Martinez-Laorden A., Arraiz-Fernandez C., Cantalejo M.J., Gonzalez-Fandos E. (2024). Prevalence, Identification and Antimicrobial Resistance of *Listeria monocytogenes* and *Listeria* spp. Isolated from Poultry and Pork Meat. Int. J. Food Sci. Technol..

[54] Olaimat A.N., Al-Holy M.A., Shahbaz H.M., Al-Nabulsi A.A., Abu Ghoush M.H., Osaili T.M., Ayyash M.M., Holley R.A. (2018). Emergence of Antibiotic Resistance in *Listeria monocytogenes* Isolated from Food Products: A Comprehensive Review. Compr. Rev. Food Sci. Food Saf..

[55] De Gaetano G.V., Lentini G., Famà A., Coppolino F., Beninati C. (2023). Antimicrobial Resistance: Two-Component Regulatory Systems and Multidrug Efflux Pumps. Antibiotics.

[56] van de Merwe C., Simpson D.J., Qiao N., Otto S.J.G., Kovacevic J., Gänzle M.G., McMullen L.M. (2024). Is the persistence of *Listeria monocytogenes* in food processing facilities and its resistance to pathogen intervention linked to its phylogeny?. Appl. Environ. Microbiol..

[57] Moura A., Leclercq A., Vales G., Tessaud-Rita N., Bracq-Dieye H., Thouvenot P., Madec Y., Charlier C., Lecuit M. (2024). Phenotypic and Genotypic Antimicrobial Resistance of *Listeria monocytogenes*: An Observational Study in France. Lancet Reg. Health Eur..

[58] Zhang J., Hao J., Wang J., Li H., Zhao D. (2025). Strategic manipulation of biofilm dispersion for controlling Listeria monocytogenes infections. Crit. Rev. Food Sci. Nutr..

[59] Hua Z., Zhu M.J. (2024). Comprehensive Strategies for Controlling *Listeria monocytogenes* Biofilms on Food-Contact Surfaces. Compr. Rev. Food Sci. Food Saf..

[60] Liu X., Xia X., Liu Y., Li Z., Shi T., Zhang H., Dong Q. (2024). Recent Advances on the Formation, Detection, Resistance Mechanism, and Control Technology of *Listeria monocytogenes* Biofilm in Food Industry. Food Res. Int..

[61] Banerji R., Karkee A., Kanojiya P., Patil A., Saroj S.D. (2020). *Listeria monocytogenes* Virulence, Antimicrobial Resistance and Environmental Persistence: A Review. Pathogens.

[62] Banerji R., Karkee A., Kanojiya P., Patil A., Saroj S.D. (2022). Bacterial Communication in the Regulation of Stress Response in *Listeria monocytogenes*. LWT.

[63] (2015). Microbiology of the Food Chain-Sampling Techniques for Microbiological Analysis of Food and Feed Samples.

[64] (2017). Microbiology of the Food Chain—Horizontal Method for the Detection and Enumeration of Listeria monocytogenes and of Listeria spp.—Part 1: Detection Method.

[65] European Committee on Antimicrobial Susceptibility Testing (2026). Clinical Breakpoint Tables for Interpretation of MICs and Zone Diameters.

[66] Magiorakos A.-P., Srinivasan A., Carey R.B., Carmeli Y., Falagas M.E., Giske C.G., Harbarth S., Hindler J.F., Kahlmeter G., Olsson-Liljequist B. (2012). Multidrug-Resistant, Extensively Drug-Resistant and Pandrug-Resistant Bacteria: An International Expert Proposal for Interim Standard Definitions for Acquired Resistance. Clin. Microbiol. Infect..

[67] Iwu C.D., Okoh A.I. (2020). Characterization of Antibiogram Fingerprints in *Listeria monocytogenes* Recovered from Irrigation Water and Agricultural Soil Samples. PLoS ONE.

[68] Morvan A., Moubareck C., Leclercq A., Hervé-Bazin M., Bremont S., Lecuit M., Courvalin P., Le Monnier A. (2010). Antimicrobial Resistance of *Listeria monocytogenes* Strains Isolated from Humans in France. Antimicrob. Agents Chemother..

